# A Five‐Year Journey to Diagnosis: Resolving Persistent Hypoglycemia Through Successful Insulinoma Resection—A Case Report

**DOI:** 10.1002/ccr3.70359

**Published:** 2025-03-21

**Authors:** Ankit Shrestha, Anup Pandey, Paleswan Joshi Lakhey, Biraj Baral, Aakash Pandit, Achyut Marahatta, Amisha Seth

**Affiliations:** ^1^ Department of Internal Medicine Chitwan Medical College Bharatpur Nepal; ^2^ Department of Internal Medicine, Maharajgunj Medical Campus, Institute of Medicine Tribhuvan University Kathmandu Nepal; ^3^ Department of Surgical Gastroenterology, Maharajgunj Medical Campus, Institute of Medicine Tribhuvan University Kathmandu Nepal

**Keywords:** distal pancreatectomy, hyperinsulinemic hypoglycemia, insulinoma, neuroendocrine tumor, neuroglycopenia

## Abstract

Insulinoma is a rare functional pancreatic neuroendocrine tumor with an annual prevalence of 0.5–5 cases per million. It is characterized by excessive insulin secretion, leading to recurrent hypoglycemia, often diagnosed through Whipple's triad: hypoglycemic symptoms, documented low plasma glucose, and symptom resolution after glucose administration. Approximately 90% of insulinomas are sporadic, while 10% are associated with multiple endocrine neoplasia type 1. Diagnosis is frequently delayed due to nonspecific symptoms and misattributions to neurological or psychiatric conditions. Biochemical confirmation through a supervised fasting test and advanced imaging modalities, including CT, MRI, and endoscopic ultrasound (EUS), is essential for identifying and localizing the tumor. We report the case of a 52‐year‐old male who presented with a 5‐year history of recurrent fasting and postprandial hypoglycemic episodes, including adrenergic and neuroglycopenic symptoms such as palpitations, diaphoresis, dizziness, and episodes of altered sensorium. Initial evaluations misattributed his symptoms to neurological and cardiac disorders, delaying diagnosis. Upon presentation, Whipple's triad was confirmed, and biochemical testing revealed hyperinsulinemic hypoglycemia (plasma glucose: 27 mg/dL, serum insulin: 45.83 mIU/L, C‐peptide: 7.03 ng/mL). Imaging identified a 3 × 3 cm hypervascular lesion in the pancreatic tail. The patient underwent distal pancreatectomy, and histopathological analysis confirmed a grade 1 neuroendocrine tumor. Postoperative outcomes were favorable, with complete resolution of symptoms and normalization of glucose and insulin levels. Follow‐up showed no recurrence of hypoglycemia. This case underscores the challenges in diagnosing insulinoma due to nonspecific symptoms and highlights the importance of Whipple's triad, biochemical tests, and imaging in timely diagnosis. Surgical resection remains the definitive treatment, with excellent long‐term outcomes when performed promptly.

**Summary**

- Early recognition of insulinoma is crucial to prevent life‐threatening hypoglycemia.
- Persistent unexplained hypoglycemia warrants evaluation for hyperinsulinemic hypoglycemia using Whipple's triad and biochemical tests.
- Advanced imaging aids tumor localization.
- Surgical resection is curative in most cases, emphasizing the importance of timely diagnosis to avoid delayed treatment and associated complications.

## 1 Introduction

Insulinoma is a very uncommon type of pancreatic endocrine tumor that causes excessive secretion of insulin within the body. It has a prevalence of 0.5–5 cases per million in the general population [1]. While 90% of insulinomas occur sporadically, the remaining 10% can be associated with multiple endocrine neoplasia type 1 [2]. Insulinomas are the primary type of functional endocrine tumor found in the pancreas. Since the clinical features of insulinoma are not specific, the diagnosis can be difficult and it heavily relies on a comprehensive patient and collateral history, along with a strong suspicion of the condition. A diagnosis of insulinoma can be suggested by Whipple's triad, which involves the presence of hypoglycemic symptoms, documented hypoglycemia, and the resolution of symptoms following glucose administration [3]. The gold standard for diagnosing hyperinsulinism involves a 72‐h supervised fasting test with measurement of plasma glucose, insulin, C‐peptide, and proinsulin levels. This biochemical confirmation method is still widely used [4]. To prevent life‐threatening hypoglycemia, it is crucial to diagnose insulinoma early. In a survey of 1928 patients with neuroectodermal tumors worldwide, it was found that there is an average delay of 52 months from the onset of symptoms to diagnosis. The study also revealed that patients consult an average of six healthcare providers before getting the accurate diagnosis. After a diagnosis of insulinoma has been established, non‐invasive imaging techniques such as computed tomography (CT) and magnetic resonance imaging (MRI) of the abdomen are employed to identify the origin of the abnormal insulin secretion [5, 6]. Endoscopic ultrasonography is an invasive technique that is highly accurate in locating insulinomas before surgery. It has been demonstrated to be more effective than non‐invasive localization methods [7]. Herein, we present the case of a 52‐year‐old male who had a 5‐year delay before diagnosing insulinoma after being initially assessed for central nervous system abnormalities.

## 2 Case History/Examination

52 year old male without history of diabetes and alcohol use disorder with no known systemic illness and not under any medication presented to the emergency department with history of recurrent fasting as well as postprandial hypoglycemic episodes within (3–4) hours after meals for 5 years. On the same day, he presented to the emergency department at a local hospital with a history of altered sensorium (somnolent type) which was preceded by adrenergic symptoms at 3 am in the morning. His glucometer random blood sugar level was documented to be 30 mg/dL, and reversal of symptoms was observed after dextrose administration, suggestive of completion of Whipple's triad. He has been having similar symptoms for the last 5 years with multiple episodes of hypoglycemic episodes per day leading to frequent eating and subsequent weight gain of 25 kg in 5 years. At admission, the patient's height was 1.63 m, and his weight was 100 kg, resulting in a BMI of approximately 37.6 kg/m^2^.

Five years back he presented to the emergency department of another tertiary care center with complaints of palpitation, diaphoresis, and dizziness followed by fainting. He was then admitted and assessed at the medicine ward of the same hospital for 1 week with conservative management after which he was discharged. In our center, He was admitted to the endocrinology ward and serum insulin, C‐peptide level, and urinary ketones were sent after inducing fasting hypoglycemia.

## 3 Methods (Investigations and Treatment)

**FIGURE 1:**
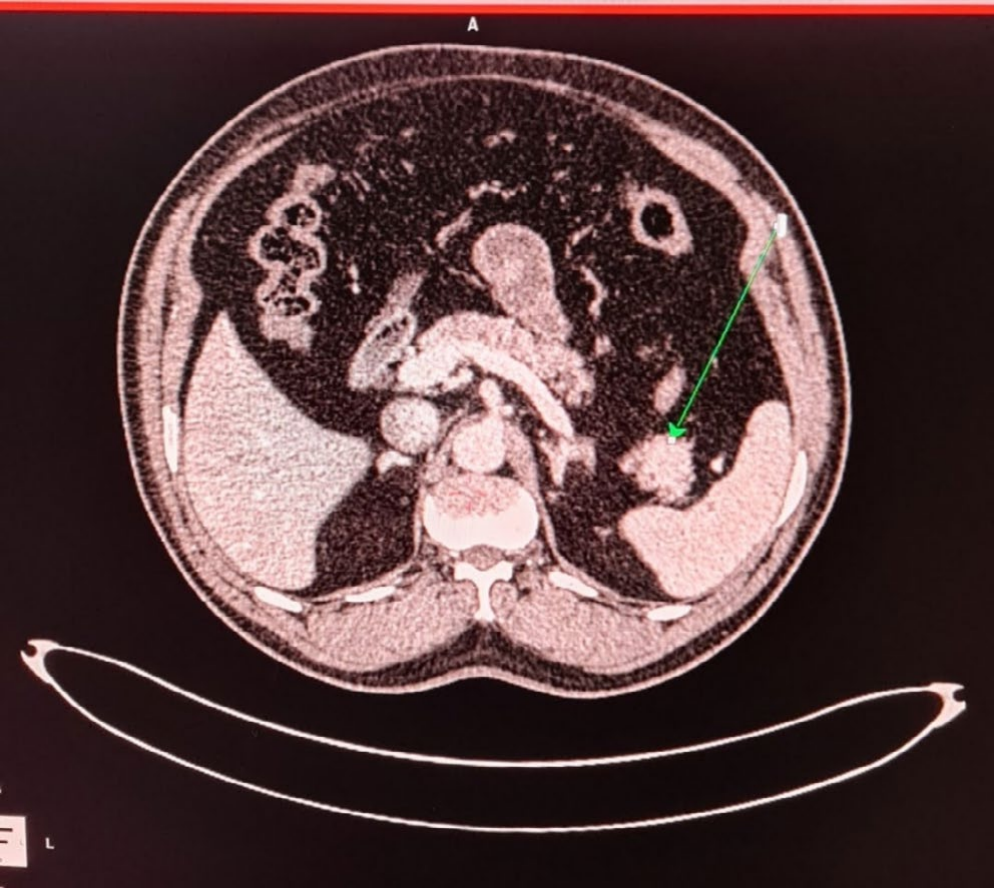
Contrast‐enhanced computed tomography of the abdomen. Arrow showing solid, slightly arterial phase hyper‐enhancing nodules within the tail of the pancreas with a small inferior exophytic component abutting branches of the splenic vein and artery superiorly.

At a venous blood glucose level of 27 mg/dL, measured 5 h after his last meal, his serum insulin was 45.83 mIU/L (2.6–37.6). C‐peptide was 7.03 ng/mL (0.48–5.05) with negative urinary ketones suggestive of hyperinsulinemic hypoglycemia. Insulin was inappropriately high for the glucose, as it should have been undetectable with a glucose level of mg/dL, with normal C‐peptide levels in conjunction with very low plasma glucose levels strongly indicating the presence of an insulinoma. His serum calcium was 8.59 mg/dL (8–11) and thyroid stimulating hormone (TSH) 0.61 microIU/ml (0.35–5.50). All other hematological and biochemical parameters were unremarkable. There is no history to suggest multiple endocrine neoplasia type 1 (no features suggestive of functional pituitary tumor, visual disturbances with normal calcium level). Contrast‐enhanced CT of the abdomen and pelvis was done to rule out pancreatic solid tumors, which showed solid, slightly arterial phase hyper‐enhancing nodules within the tail of the pancreas, with a small inferior exophytic component abutting branches of the splenic vein and artery superiorly (Figure 1).

EUS showed the presence of an ill‐defined hypoechoic hypo‐vascular lesion in the tail of the pancreas which is in close relation with splenic vessels but not abutting splenic vessels.

**FIGURE 2:**
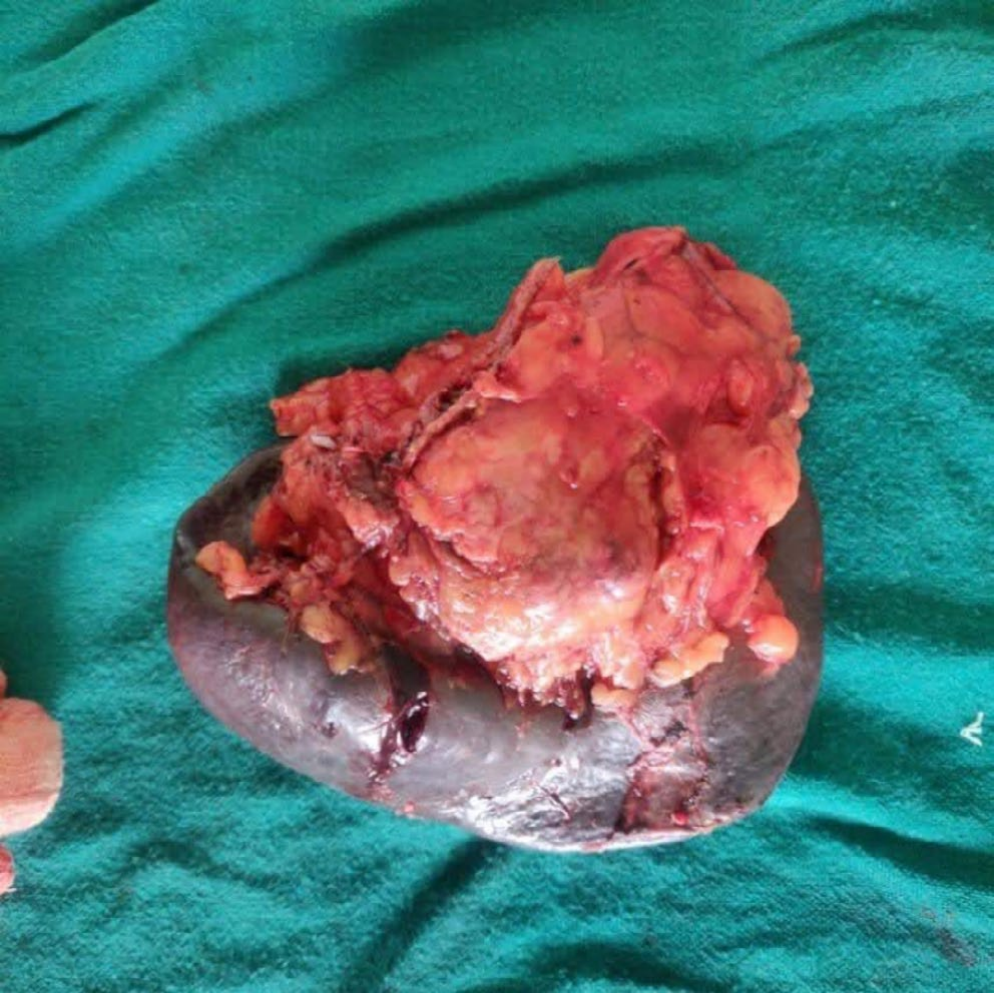
Resected specimen consisting of part of the pancreas measuring (11 × 7 × 2) cm and spleen measuring (13 × 7 × 4.5) cm.

Standard Distal Pancreatectomy was done with removal of about 3 × 3 cm firm mass at the tail of pancreas without peripancreatic lymph node involvement and without splenic and adjacent tissue invasion (Figure 2).

**FIGURE 3:**
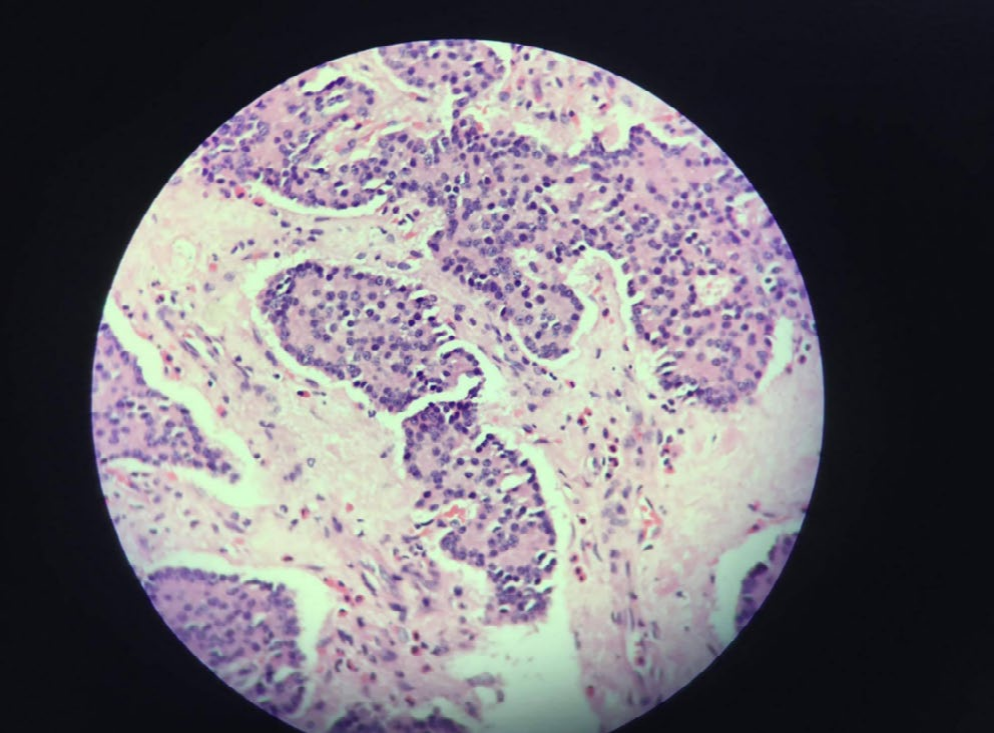
Microscopic image of the biopsy of the resected tissue stained with Hematoxylin and Eosin (H&E) and examined at 100× magnification, showing a well‐differentiated neuroendocrine tumor, grade 1 (G1).

The histopathological examination of the pancreatic tail lesion confirmed it as a well‐differentiated neuroendocrine tumor, grade 1 (G1) (Figure 3). Importantly, when the spleen was examined microscopically, no evidence of malignant cells, tumor infiltration, or metastasis was found. This means that the spleen's normal architecture was preserved, indicating that the tumor was confined to the pancreas and had not spread to the spleen.

## 4 Conclusion and Results (Outcome and Follow‐Up)

Following the surgery, the neuroglycopenic symptoms and altered sensorium were resolved. On a 4‐week follow‐up, the fasting insulin was 5.43 mIU/L, fasting C‐peptide was 4.03 ng/mL, and fasting blood sugar (FBS) was 110 mg/dL.

## 5 Discussion

Hypoglycemic symptoms can be categorized into two main types. The first type is neuroglycopenic signs, which are the most common and include confusion, changes in behavior, problems with vision, weakness, dizziness, seizures, and loss of consciousness. The second type is neurogenic signs, which encompass symptoms like anxiety, excessive sweating, rapid heartbeats, trembling, and a sensation of warmth [8, 9].

These symptoms typically manifest when a person has been fasting and are frequently triggered by physical activity. Insulinoma, a rare yet significant cause of hypoglycemia, presents complex diagnostic challenges due to its nonspecific symptoms. In this case of a 52‐year‐old male with prolonged hypoglycemia over 5 years, the delayed diagnosis reflects a common trend for insulinomas. Studies indicate that the average delay from symptom onset to diagnosis can reach 4 years, as insulinomas are often misinterpreted as neurologic or psychiatric disorders, leading to misdiagnosis and inappropriate treatment paths. An agnostic journey often results in multiple provider consultations before reaching an accurate diagnosis, highlighting a need for increased awareness of insulinoma's distinct clinical features [10].

In our report, insulinoma diagnosis was delayed for 5 years after the symptoms started, Simply because symptoms of hypoglycemia have been misinterpreted and misattributed to cardiac and neurological disorders before the insulinoma was recognized. In addition, our patients tried to avoid hypoglycemic signs by eating frequently with resultant weight gain.

The diagnosis of insulinoma is anchored in demonstrating hyperinsulinemic hypoglycemia during a supervised fasting test, where prolonged fasting triggers hypoglycemia, allowing measurement of plasma insulin, C‐peptide, and proinsulin levels. In this case, blood glucose was at hypoglycemic levels just 5 h after the last meal—an early onset that underscores the unregulated insulin secretion from the tumor and supports the diagnosis of insulinoma. Elevated insulin alongside low plasma glucose without ketosis remains a defining feature of insulinoma and serves as a critical indicator in differentiating insulinoma from other hypoglycemic etiologies such as reactive hypoglycemia and adrenal insufficiency [9].

Fasting testing in patients with suspected hypoglycemia should be performed in a controlled, inpatient setting—typically via a prolonged (up to 72‐h) fast or until hypoglycemia is documented. During the fast, serial blood samples are obtained to measure plasma glucose, insulin, C‐peptide, and proinsulin levels, which are critical for detecting inappropriate insulin secretion. In parallel, measuring ketone bodies (especially β‐hydroxybutyrate) is important because low ketone levels during hypoglycemia indicate persistent insulin or insulin‐like hormone activity that suppresses lipolysis and ketogenesis. At the end of the fast, an intravenous glucagon challenge is often performed; an appropriate rise in plasma glucose following glucagon administration confirms that hepatic glycogen stores are preserved and supports the diagnosis of an insulinoma versus other hypoglycemic conditions such as reactive hypoglycemia or adrenal insufficiency [11].

Some insulinomas present exclusively with postprandial hypoglycemia, meaning that affected patients may experience low blood sugar symptoms only after meals rather than during fasting. In these cases, the tumor's insulin secretion is triggered predominantly by nutrient intake, leading to an exaggerated postprandial insulin response that causes hypoglycemia. This atypical pattern can delay diagnosis, as the absence of fasting hypoglycemia might lead clinicians to initially rule out insulinoma. For example, Placzkowski et al. (2009) reported that a subset of insulinoma patients exhibited solely postprandial hypoglycemic episodes, and classical descriptions emphasize that while fasting hypoglycemia is common, its absence does not exclude insulinoma if postprandial hypoglycemia is present. Recognizing this variant is critical to ensuring timely diagnosis and appropriate management [12].

Some individuals develop hypoglycemia unawareness, a condition in which repeated episodes of low blood glucose blunt the normal autonomic warning symptoms (such as sweating, tremor, and palpitations). As a result, these patients may present initially with neuroglycopenic symptoms—like confusion, impaired cognition, or even seizures—without prior warning, which can delay recognition and treatment of hypoglycemia [13].

Medical management plays a critical role for patients with insulinoma who are not candidates for surgery or who have recurrent or metastatic disease. For instance, diazoxide—a potassium channel opener that inhibits insulin secretion from pancreatic β‐cells—is widely used as a first‐line agent, although its use may be limited by side effects such as fluid retention and hirsutism. In addition, somatostatin analogues (e.g., octreotide and lanreotide) can suppress hormone release, and newer agents such as everolimus (an mTOR inhibitor) and pasireotide (a multireceptor somatostatin analogue) have shown promise in controlling refractory hypoglycemia. These therapies expand the treatment options available to achieve symptomatic control and tumor stabilization [14].

Imaging technique plays a pivotal role in localizing insulinomas preoperatively. Non‐invasive modalities, such as CT and MRI, are first‐line choices for visualization, while EUS is employed for higher precision in localization, particularly for small pancreatic lesions. In this case, CT identified a nodule in the pancreatic tail, while EUS further clarified the lesion's proximity to splenic vessels without direct vascular involvement. Studies have demonstrated that the sensitivity in detecting insulinomas is as high as 90%, especially for lesions under 2 cm, underscoring its value in preoperative planning and reducing the risk of intraoperative complications [10].

Surgical resection remains the treatment for insulinomas, with distal pancreatectomy favored for lesions in the pancreatic tail. This approach is curative in most cases, with studies indicating a long‐term survival rate of over 95% when complete resection is achieved [15]. Our patient's distal pancreatectomy resulted in the resolution of hypoglycemic symptoms and normalized insulin and glucose levels, consistent with the curative outcomes reported in the literature [16]. Furthermore, histopathology confirmed a well‐differentiated neuroendocrine tumor, or G1 insulinoma, without malignancy, aligning with the typically benign nature of most insulinomas [10]. In cases where malignant features are absent, postoperative management primarily focuses on monitoring for recurrence and managing metabolic health [15].

In addition to surgical intervention, alternative approaches have been reported for the management of benign insulinomas. These include the administration of octreotide via injection, EUS‐guided alcohol ablation, radio frequency ablation (RFA), or embolization techniques specifically targeted at insulinomas in the pancreas. The medical management of insulinoma, aimed at treating and preventing hypoglycemia, is typically limited to certain situations. These include cases where the tumor cannot be surgically removed due to metastasis, instances where surgery has been unsuccessful in relieving persistent symptoms, patients who are not eligible for surgery, and individuals who are either waiting for surgery or decline surgical intervention [17].

An essential aspect of this case is the weight gain experienced by the patient over the years, attributable to compensatory eating to prevent hypoglycemic episodes. This phenomenon, which often goes unrecognized, can provide clues to an underlying insulinoma, as patients attempt to alleviate symptoms by frequent food intake. Left untreated, insulinoma‐induced hyperphagia can lead to significant weight gain and metabolic complications, as seen in this case, which further emphasizes the importance of early diagnosis to avoid such adverse outcomes [9]. Comprehensive post‐surgical management, including nutritional guidance, is crucial for restoring metabolic stability and addressing any residual weight or glycemic concerns [15].

## 6 Conclusion

This case of insulinoma exemplifies the significance of vigilance and precision in diagnosing rare causes of recurrent hypoglycemia. Despite the patient's extended journey through multiple healthcare consultations, the ultimate identification of insulinoma via Whipple's triad and advanced imaging led to a successful surgical intervention, marking a complete resolution of symptoms. The case underscores the critical role of a timely diagnosis—not only to alleviate immediate symptoms but to prevent life‐altering consequences of untreated hypoglycemia.

## Author Contributions

**Ankit Shrestha:** conceptualization, data curation, formal analysis, investigation, methodology, project administration, resources, supervision, validation, visualization, writing – original draft, writing – review and editing. **Anup Pandey:** conceptualization, investigation, methodology, resources, supervision, validation, visualization, writing – original draft, writing – review and editing. **Paleswan Joshi Lakhey:** conceptualization, data curation, formal analysis, investigation, methodology, resources, software, validation, visualization. **Biraj Baral:** conceptualization, data curation, investigation, methodology, supervision, validation. **Aakash Pandit:** validation, writing – review and editing. **Achyut Marahatta:** writing – review and editing. **Amisha Seth:** writing – review and editing.

## Consent

Written informed consent was obtained from the patient for publication and any accompanying images.

## Conflicts of Interest

The authors declare no conflicts of interest.

## Data Availability

The data that support the findings of this study are available from the corresponding author upon reasonable request.

## References

[1] Z. Qi , D. Li , J. Ma , P. Xu , Y. Hao , and A. Zhang , “Insulinoma Presenting as a Complex Partial Seizure: Still a Possible Misleading Factor,” Frontiers in Neuroscience 13 (2020): 1388, 10.3389/fnins.2019.01388. 32009878 PMC6978910

[2] S. L. Asa , “Pancreatic endocrine tumors,” Modern Pathology 24, no. 2 (2011): S66–S77, 10.1038/modpathol.2010.127. 21455203

[3] P. R. Nashidengo , F. W. Quayson , J. T. Abebrese , L. Negumbo , C. Enssle , and F. Kidaaga , “Varied Presentations of Pancreatic Insulinoma: A Case Report,” Pan African Medical Journal 42 (2022): 69, 10.11604/pamj.2022.42.69.34839. 35949463 PMC9338700

[4] T. Okabayashi , Y. Shima , T. Sumiyoshi , et al., “Diagnosis and Management of Insulinoma,” World Journal of Gastroenterology 19, no. 6 (2013): 829–837, 10.3748/wjg.v19.i6.829. 23430217 PMC3574879

[5] T. C. Noone , J. Hosey , Z. Firat , and R. C. Semelka , “Imaging and Localization of Islet‐Cell Tumours of the Pancreas on CT and MRI,” Best Practice & Research. Clinical Endocrinology & Metabolism 19, no. 2 (2005): 195–211, 10.1016/j.beem.2004.11.013. 15763695

[6] S. Singh , D. Granberg , E. Wolin , et al., “Patient‐Reported Burden of a Neuroendocrine Tumor (NET) Diagnosis: Results From the First Global Survey of Patients With NETs,” JCO Global Oncology 3, no. 1 (2017): 43–53, 10.1200/jgo.2015.002980. PMC5493232 28717741

[7] P. H. Kann , M. Rothmund , and A. Zielke , “Endoscopic Ultrasound Imaging of Insulinomas: Limitations and Clinical Relevance,” Experimental and Clinical Endocrinology & Diabetes: Official Journal, German Society of Endocrinology 113, no. 8 (2005): 471–474, 10.1055/s-2005-865752. 16151982

[8] B. Abboud and J. Boujaoude , “Occult Sporadic Insulinoma: Localization and Surgical Strategy,” World Journal of Gastroenterology 14, no. 5 (2008): 657–665, 10.3748/wjg.14.657. 18205253 PMC2683990

[9] P. E. Cryer , “Symptoms of Hypoglycemia, Thresholds for Their Occurrence, and Hypoglycemia Unawareness,” Endocrinology and Metabolism Clinics of North America 28, no. 3 (1999): 495–500, 10.1016/s0889-8529(05)70084-0. 10500927

[10] C. S. Grant , “Insulinoma,” Best Practice & Research. Clinical Gastroenterology 19, no. 5 (2005): 783–798, 10.1016/j.bpg.2005.05.008. 16253900

[11] M. S. Rayas and M. Salehi , “Non‐Diabetic Hypoglycemia,” in Endotext, ed. K. R. Feingold , B. Anawalt , M. R. Blackman , et al. (MDText.com, Inc., 2024), https://www.ncbi.nlm.nih.gov/books/NBK355894/.

[12] D. Vezzosi , A. Bennet , J. C. Maiza , et al., “Diagnosis and Treatment of Insulinomas in the Adults,” in Basic and Clinical Endocrinology Up‐to‐Date (InTech, 2011), 10.5772/17452.

[13] P. E. Cryer , S. N. Davis , and H. Shamoon , “Hypoglycemia in Diabetes,” Diabetes Care 26, no. 6 (2003): 1902–1912, 10.2337/diacare.26.6.1902. 12766131

[14] E. Brown , D. Watkin , J. Evans , V. Yip , and D. J. Cuthbertson , “Multidisciplinary Management of Refractory Insulinomas,” Clinical Endocrinology 88, no. 5 (2018): 615–624, 10.1111/cen.13528. 29205458

[15] J. N. Fahmy , M. A. Varsanik , D. Hubbs , E. Eguia , G. Abood , and L. M. Knab , “Pancreatic Neuroendocrine Tumors: Surgical Outcomes and Survival Analysis,” American Journal of Surgery 221, no. 3 (2021): 529–533, 10.1016/j.amjsurg.2020.12.037. 33375953

[16] F. J. Service , M. M. McMahon , P. C. O'Brien , and D. J. Ballard , “Functioning Insulinoma—Incidence, Recurrence, and Long‐Term Survival of Patients: A 60‐Year Study,” Mayo Clinic Proceedings 66, no. 7 (1991): 711–719, 10.1016/s0025-6196(12)62083-7. 1677058

[17] J. Hofland , J. C. Refardt , R. A. Feelders , E. Christ , and W. W. de Herder , “Approach to the Patient: Insulinoma,” Journal of Clinical Endocrinology and Metabolism 109, no. 4 (2024): 1109–1118, 10.1210/clinem/dgad641. 37925662 PMC10940262

